# Social profiles project - only the strong survive

**DOI:** 10.1186/1750-1172-9-S1-O32

**Published:** 2014-11-11

**Authors:** Birthe Byskov Holm

**Affiliations:** 1 Rare Diseases Denmark, 2630 Høje Taastrup, Denmark

## Introduction

Rare Diseases Denmark has developed a tool, Social Profiles, to promote the dialogue between the rare disease patients and their social workers conducted. This is more necessary than ever - an interview study during the fall 2013 shows substantial problems in the interaction between the families and the health care sector and social services.

## Method

The Social Profiles was developed with the involvement of volunteers from the member associations of Rare Diseases Denmark. The interview study included parents, adolescents and adults as well as adults with rare diseases were interviewed. The interviews were semi-structured, and analyzed on the basis of grounded theory.

## Results from interview study

The interviews showed that you must be resourceful to be able to navigate the welfare systems, as one mother said: ‘only the strong survive’.

The core problems in the health care sector were described as challenges not being taken seriously, difficulties in being diagnosed and referred to the right specialists. Lack of coordination and overview are key problems for adult patients, who are not affiliated to the national centers of rare diseases.

One of the main problems for the rare disease patients meeting the social service sector is the startup phase. Many families discovered until late the opportunity to receive social support. Furthermore families are experiencing a lack of transparency in the case-management. The rarity leads to increased documentation requirements, which can be difficult because of the lack of knowledge of rare diseases and because the standard procedures are inadequate. The rare families encounter distrust among caseworkers because of the lack of knowledge of the diagnosis. Therefore they feel distrusted and experience a cumbersome system with slow processing of applications and numerous appeals.

## Initiatives – Social Profiles

Rare Diseases Denmark has developed a tool, Social Profiles, to promote the dialogue between the rare disease patients and their social workers. The Social Profiles bring the professionals up to speed on their knowledge about the diagnosis, its symptoms, treatment, prognosis and variation. These characteristics are verified by a physician.

The Social Profiles also provide a checklist with special needs, which differ according to the age of the rare disease patient. It also offers references to patient societies and patient associations.

The Social Profiles are developed by the patient associations and hosted on the website: http://www.sjaeldenborger.dk.

